# *Paracoccidioides brasiliensis* Releases a DNase-Like Protein That Degrades NETs and Allows for Fungal Escape

**DOI:** 10.3389/fcimb.2020.592022

**Published:** 2021-02-10

**Authors:** Yohan Ricci Zonta, Ana Laura Ortega Dezen, Amanda Manoel Della Coletta, Kaio Shu Tsyr Yu, Larissa Carvalho, Leandro Alves dos Santos, Igor de Carvalho Deprá, Rachel M. Kratofil, Michelle Elizabeth Willson, Lori Zbytnuik, Paul Kubes, Valdecir Farias Ximenes, Luciane Alarcão Dias-Melicio

**Affiliations:** ^1^ Laboratory of Immunopathology and Infectious Agents - LIAI, UNIPEX - Experimental Research Unity, Sector 5, Medical School of Botucatu, São Paulo State University (UNESP), Botucatu, Brazil; ^2^ Confocal Microscopy Laboratory, UNIPEX - Experimental Research Unity, Medical School of Botucatu, São Paulo State University (UNESP), Botucatu, Brazil; ^3^ Laboratory of Genetic Basis of Endocrinological Diseases, Experimental Research Unity (UNIPEX), Sector 5, São Paulo State University (UNESP), Botucatu, Brazil; ^4^ Calvin, Phoebe, and Joan Snyder Institute for Chronic Diseases, University of Calgary, Calgary, AB, Canada; ^5^ Department of Physiology and Pharmacology, Cumming School of Medicine, University of Calgary, Calgary, AB, Canada; ^6^ Department of Microbiology, Immunology and Infectious Diseases, Cumming School of Medicine, University of Calgary, Calgary, AB, Canada; ^7^ Department of Chemistry, Sciences School, São Paulo State University (UNESP), Bauru, Brazil; ^8^ Department of Pathology, Medical School of Botucatu, São Paulo State University (UNESP), Botucatu, Brazil

**Keywords:** paracoccidioidomycosis, neutrophils, neutrophil extracellular traps (NETs), DNase, escape mechanism

## Abstract

Paracoccidioidomycosis is a systemic fungal disease, considered endemic in Latin America. Its etiological agents, fungi of the *Paracoccidioides* complex, have restricted geographic habitat, conidia as infecting form, and thermo-dimorphic characteristics. Polymorphonuclear neutrophils (PMNs) are responsible for an important defense response against fungus, releasing Neutrophil Extracellular Traps (NETs), which can wrap and destroy the yeasts. However, it has been described that some pathogens are able to evade from these DNA structures by releasing DNase as an escape mechanism. As different NETs patterns have been identified in PMNs cultures challenged with different isolates of *Paracoccidioides brasiliensis*, the general objective of this study was to identify if different patterns of NETs released by human PMNs challenged with Pb18 (virulent) and Pb265 (avirulent) isolates would be correlated with fungal ability to produce a DNase-like protein. To this end, PMNs from healthy subjects were isolated and challenged *in vitro* with both fungal isolates. The production, release, and conformation of NETs in response to the fungi were evaluated by Confocal Microscopy, Scanning Microscopy, and NETs Quantification. The identification of fungal DNase production was assessed by DNase TEST Agar, and the relative gene expression for hypothetical proteins was investigated by RT-qPCR, whose genes had been identified in the fungal genome in the GenBank (PADG_11161 and PADG_08285). It was possible to verify the NETs release by PMNs, showing different NETs formation when in contact with different isolates of the fungus. The Pb18 isolate induced the release of looser, larger, and more looking like degraded NETs compared to the Pb265 isolate, which induced the release of denser and more compact NETs. DNase TEST Agar identified the production of a DNase-like protein, showing that only Pb18 showed the capacity to degrade DNA in these plates. Besides that, we were able to identify that both PADG_08528 and PADG_11161 genes were more expressed during interaction with neutrophil by the virulent isolate, being PADG_08528 highly expressed in these cultures, demonstrating that this gene could have a greater contribution to the production of the protein. Thus, we identified that the virulent isolate is inducing more scattered and loose NETs, probably by releasing a DNase-like protein. This factor could be an important escape mechanism used by the fungus to escape the NETs action.

## Introduction

Paracoccidioidomycosis (PCM) is a systemic fungal disease, considered a neglected disease endemic to Latin America, occurring from Mexico to Argentina with its main foci of infection in countries such as Brazil, Venezuela, and Colombia, usually affecting agricultural workers, gardeners, and transporters (Coutinho et al., 2002; Restrepo and Tobón, 2005; Colombo et al., 2011; Marques, 2013). Some cases have been reported outside the endemic areas, such as Europe and North America, but in these cases, the infected individuals moved to these regions after infection occurred (Wagner et al., 2016). Its etiological agents are fungi from the *Paracoccidioides* complex, composed of five species that are restricted geographically (Matute et al., 2006). The infectious form is conidia with thermo-dimorphic characteristics, which means that it alters its morphology according to the temperature to which it is exposed, taking the form of mycelium at room temperature, and upon entering the body (37°C), it turns into yeast form (Colombo et al., 2011). The fungus *Paracoccidioides* spp. belongs to the same family as other etiological agents that cause deep fungal mycoses, such as *Histoplasma capsulatum* and *Coccidioides immitis*, that are also onygenalean human pathogenic fungi (Matute et al., 2006). A recent study used molecular polymorphism, phylogenetic reconstruction, genetic and morphological population analysis of yeasts and conidia of different species demonstrated that the *Paracoccidioides* complex (previously known as four cryptic species of the *P. brasiliensis* and *P. lutzii*) can actually be divided in different species, and with that, proposed a new nomenclature for these different agents: *P. americana* for PS2, *P. restrepiensis* for PS3, and *P. venezuelensis* for PS4, while *P. brasiliensis* would be restricted to species S1 (Matute et al., 2006; Teixeira et al., 2009; Teixeira et al., 2014; Turissini et al., 2017).

After inhalation, the infectious conidia are lodged in the lower airways (bronchioles and alveoli) (Marques, 2013; Shikanai-Yasuda et al., 2018) where they germinate into yeast forming the Primary Pulmonary Complex (Severo et al., 1979). The fungus can be destroyed or become latent, characterizing PCM-infection (Shikanai-Yasuda et al., 2018), or spread to other organs such as the liver, spleen, adrenal *via* the lymphohematogenous pathway, characterizing PCM-disease (Shikanai-Yasuda et al., 2018), which can be presented in two different forms, the acute/subacute and the chronic forms (Bocca et al., 2013; Shikanai-Yasuda et al., 2018). In the specific case of PCM, the massive infiltration of neutrophils was found in the inflammatory tissues of patients (Franco, 1987), as well as in the lesions of different experimental models of the disease (Defaveri et al., 1989; Defaveri et al., 1992; Calich et al., 1994). Human neutrophils are associated with resistance to *P. brasiliensis* infection *in vitro*, as polymorphonuclear cells can ingest the fungal yeast through the phagocytosis process (Dias et al., 2004). However, when the yeasts are larger than the supported size by the cell, the cells generate a kind of extracellular vacuole before attacking the fungus (Dias et al., 2004). However, although sometimes neutrophils are successful in the phagocytosis process, this does not seem to be sufficient to kill the fungus (Kurita et al., 1999). Some studies have shown that human neutrophils have no fungicidal or fungistatic activity against *P. brasiliensis* (Kurita et al., 1999; Kurita et al., 2005). This activity, however, is increased when cells are activated by IFN-γ, TNF-α, GM-CSF, or Reactive Oxygen Species (ROS), which initiates an offensive mechanism against the fungus dependent on reactive oxygen species (Rodrigues et al., 2007). However, studies indicate that specific cytokines, such as IL-10, also act as suppressors of polymorphonuclear mediated response (Costa et al., 2007).

A key defense mechanism of neutrophils is the ability to expel nuclear contents into the surrounding environment in the form of neutrophil extracellular traps (NETs). NETs are composed of large amounts of DNA decorated with granule proteins such as enzymes, histones, and antimicrobial peptides (Brinkmann et al., 2004; Brinkmann and Zychlinsky, 2012; Ravindran et al., 2019). The high concentration of these proteins within NETs, associated with the physical structure created around the microorganism, are directly associated with the antimicrobial activity of these structures (Brinkmann et al., 2004; Pilsczek et al., 2010; Papayannopoulos et al., 2010; McDonald et al., 2012; Ravindran et al., 2019; Thanabalasuriar et al., 2019; Castanheira and Kubes, 2019; Liew and Kubes, 2019).

Recently, it was demonstrated that *P. brasiliensis* could induce the release of NETs (Mejía et al., 2015; Della Coletta et al., 2015; Bachiega et al., 2016). Our research group identified that the virulent fungal isolate (Pb18) is able to induce NETs release by human neutrophils from healthy individuals *via* dectin-1 signaling, and that NET release resulted in extracellular fungicidal activity (Bachiega et al., 2016). Additionally, we have also identified the presence of NETs in cutaneous lesions of patients with paracoccidioidomycosis, and after challenging neutrophils from these patients with Pb18 (more virulent) and Pb265 (less virulent) isolates, we identified different patterns of NET formation induced by the different fungal isolates (Della Coletta et al., 2015). In the more virulent Pb18 isolate, the NET pattern was loose and scattered covering a large surface area, whereas in the less virulent Pb265 isolate, NETs were smaller, more dense and compacted (Della Coletta et al., 2015). We previously hypothesized that the different patterns of NETs could be due to the production of a DNase or an enzyme with DNase-like activity by the Pb 18 isolate as a mechanism of fungal escape from host defenses, leading to degradation of NETs during fungal infection (Della Coletta et al., 2015). It was shown that some pathogens such as *Staphylococcus aureus* (Berends et al., 2010; Thammavongsa et al., 2013; Hoppenbrouwers et al., 2018), *Pseudomonas aeruginosa* (Wilton et al., 2018), *Mycoplasma bovis* (Zhang et al., 2016; Gondaira et al., 2017; Mitiku et al., 2018)*, Streptococcus spp* (Buchanan et al., 2006; de Buhr et al., 2014; Morita et al., 2014; Liu et al., 2017), *Vibrio cholerae* (Seper et al., 2013)*, Ehrlichia chaffeensis* (Teymournejad et al., 2017), other bacterias (Sharma et al., 2019), and beyond, *Leishmania spp* (Freitas-Mesquita et al., 2019)*, Candida albicans* (Riceto et al., 2015; Zhang et al., 2017)*, Cryptococcus spp* (Sánchez and Colom, 2010)*, Trichosporon spp* (Bentubo and Gompertz, 2014), are able to produce DNase or exonucleases as an escape mechanism from NETs.

Thus, the objective of this work was to identify whether the fungal isolates Pb18 and Pb265 produce a DNase or DNase-like enzyme that could degrade NETs from human neutrophils *in vitro*, and correlate this enzymatic activity with the NETs patterns observed by the different fungal isolates. Ultimately, this study is fundamental to better understand the fungal mechanisms involved in the escape of host defenses.

## Materials and Methods

### Casuistics

Neutrophils were obtained from 40 ml of peripheral blood after venipuncture of 10 healthy volunteers. All subjects were informed of the research goals and signed a consent form. The study was conducted according to the National Health Guidelines 196/96, and it was approved by the Research Ethics Committee of Medical School of Botucatu, UNESP – São Paulo State University (CAAE: 85654018.9.0000.5411; protocol: 2.577.243/2018).

### Neutrophil Isolation

After the venous puncture, neutrophils were isolated by a density gradient centrifugation (Histopaque^®^ 1,119 g/ml and Histopaque^®^ 1,083 g/ml - Sigma-Aldrich - St. Louis, Missouri, USA) at 1,500 rpm for 30 min (room temperature). Erythrocytes were lysed with a hypotonic solution (NaCl 0.1%), and the neutrophil viability was assessed with trypan blue dye exclusion (95% viability). Cell cultures were then resuspended in RPMI-1640 medium (Cultilab – Campinas, São Paulo, BRA) supplemented with 10% of calf serum (Sigma-Aldrich) and 1% Gentamicin (Schering- Plough, New Jersey, EUA) and adjusted for 2x10^6^ cells/ml before all procedures.

### Fungal Isolates and Culture Conditions

Two isolates of *P. brasiliensis* were used during the experiments: Pb18, a virulent isolate, and Pb265, a non-virulent isolate. Isolates were cultivated in GPY agar (BD - Franklin Lakes, New Jersey, USA) and incubated at 37°C for six days. After the growing period, a sample was collected and diluted in RPMI-1640 (Cultilab) medium in a tube containing glass beads. The tubes were vortexed to separate de bud cells from the mother cells, and after sedimentation for 5 min, the supernatant with smaller yeasts was collected. After that, the fungi were counted in a Neubauer chamber to verify the cell concentration and viability by the phase-contrast method (Acorci et al., 2009). The fungus concentration was adjusted to 4x10^4^ cells (1:50 ratio).

### Immunofluorescence for Neutrophil Extracellular Traps Visualization and Quantification

Isolated neutrophils (500 µl/well) from healthy volunteers adhered to coverslips treated with Poly-L-Lysine 0, 01% (Sigma-Aldrich) in 24-well bottom for adherence. Cells were then challenged with 500 µl/well from *P. brasiliensis* (Pb18 and Pb265) for 2 h at 37°C in 5% of CO2. Some cultures were treated with PMA (Sigma Aldrich, 100 ng/ml) as a positive control, and some wells were treated with DNase (100 U/ml– Thermo Fisher Scientific - Waltham, Massachusetts, USA), as a negative control. Cocultures were then fixed with PBS+BSA (bovine serum albumin)+PFA (paraformaldehyde) 2% and incubated with PBS+BSA 5% (for nonspecific binding block) for 30 min. Anti-neutrophil elastase at 0,1% (Calbiochem – Merck Millipore - Burlington, Massachusetts, USA) and anti-histone H1 at 0,9% (Merck Millipore) primary antibodies were diluted in blocking buffer and added to the coverslips for 1 h a 37°C. Cultures were washed three times with PBS and incubated with anti-rabbit-FITC at 1% (Millipore) and anti-mouse-Texas red at 0,5% (Calbiochem – Merck Millipore) secondary antibodies for elastase and histone visualization. Mounting medium for fluorescence with DAPI was used to mount the slides (Vectashield-Vector Labs, Burlingame, California, USA). Confocal images were taken in a Leica TCS SP8 from Confocal Microscopy Laboratory, UNIPEX - Experimental Research Unity, FMB-UNESP, Botucatu. The images were further analyzed using IMARIS^®^ software (Oxford Instruments) at Calvin, Phoebe, and Joan Snyder Institute for Chronic Diseases, University of Calgary/CA. The total area and intensity of the NETs were analyzed using the images of cultures challenged with Pb18 and Pb265, with the analysis of the elastase staining.

### Neutrophil Extracellular Traps by Scanning Electron Microscopy

Neutrophils cultures underwent 2 h of adherence on coverslips treated with Poly-L-Lysine 0, 01% (Sigma-Aldrich) in 24-well bottom plates before fungal challenge or activation with PMA (Sigma Aldrich, 100 ng/ml). Some cultures were treated with DNase (Thermo-Fisher, 100 U/ml) as a negative control. After 2 h of challenge, supernatants were removed, and cultures were fixed with 2.5% of glutaraldehyde for scanning electron microscopy analysis at CME (Electron Microscopy Center – Institute of Bioscience — UNESP— Botucatu). The analyses were performed under a scanning microscope FEI Quanta 200 model from the same lab above.

### Neutrophil Extracellular Trap Quantification

Supernatants of neutrophils cultures challenged with Pb18, and Pb265 isolates were collected for NETs quantification by an immunoassay using anti-elastase (Calbiochem – Merck Millipore) and Quant-iT™ PicoGreen^®^ kit (Invitrogen - Carlsbad, Califórnia, USA) according to the manufacturer’s instructions, with some adaptations (Czaikoski et al., 2016; Colón et al., 2019). Some cultures were treated with PMA or DNase, as positive and negative controls, respectively. Briefly, anti-elastase (5 µg/ml) antibody (Calbiochem – Merck Millipore) was used to coat a 96-well clear-bottom black plate (Corning - Corning, Nova York, EUA) overnight at 4°C. Supernatants were then settled to the plate, and the extracellular DNA bounded to the elastase, a NET constituent, was measured with Quant-iT™ PicoGreen^®^ kit to evaluate and to quantify these complexes. Samples were analyzed by fluorescence intensity (excitation at 488 nm and emission at 525 nm wavelength) by a FlexStation 3 Microplate Reader (Molecular Devices, CA, USA), and concentrations were calculated comparing to a standard curve using known levels of DNA concentrations.

### DNase Test Agar

To identify the possible DNase-like production, with an extracellular deoxyribonuclease activity, we utilized a DNase Test Agar (Acumedia, Neogen Culture Media) supplemented with 0,5% gentamicin sulfate (Novafarma) and 5% fungal aqueous extract (obtained from our Pb192 isolate). Petri plates (90x15 mm) were filled with 25 ml of medium and were incubated for one day before the experiment to exclude any contaminated plate. Each of the 4x10^4^ cells/ml fungal suspension was diluted until 4x10^3^ cells/ml and had 100 μl plated using an L-shaped glass rod. The plates were flooded with HCl 1N to precipitate the DNA, after 14 days of incubation, then a halo around the colonies with some deoxyribonuclease activity was revealed. As a positive and negative control, *Staphylococcus aureus and Saccharomyces cerevisiae*, respectively, were also plated in the DNase Test Agar medium, showing the effectiveness of the method in identifying positive DNase colonies.

### Real-Time RT-PCR

To verify the gene expression related to a possible DNase-like production, we performed a Real-Time RT-PCR to verify the mRNA expression of two potential genes related, PADG_11161 and PADG_08285, according to GenBank. At first, neutrophil cultures were challenged with both isolates of *P. brasiliensis*, as previously mentioned, in the periods of 0 and 2 h in the incubator at 37°C in 5% CO2. The cells were lysed with TRI Reagent^®^ RNA Isolation Reagent (Sigma-Aldrich, San Luis, Missouri, EUA), and the mRNA was extracted utilizing the Invitrogen™ PureLink™ RNA Mini Kit according to the manufacturer´s instruction (Invitrogen, Carlsbad, Califórnia, EUA). The mRNA samples were treated with DNase I, RNAse free (Thermo Fisher ScientificTM Waltham, Massachusetts, EUA) and a ribonuclease inhibitor (Thermo Fisher Scientific) and then transformed into cDNA by reverse transcription using the High-Capacity RNA-to-cDNA™ Kit (Applied Biosystems™, Foster City, Califórnia, EUA). The Real-Time RT-PCR was performed on the StepOnePlus™ Real-Time PCR System (Applied Biosystems™) with LUNA™ Universal qPCR Master Mix (New England Biolabs Inc., Ipswich, Massachusetts, EUA). The hypothetical sequences responsible for the deoxyribonuclease production were obtained from GenBank (https://www.ncbi.nlm.nih.gov/genbank/) and the primers, designed on Primer-BLAST (Ye et al., 2012) – see **Table 1**. Reactions were prepared with Luna Universal qPCR Master Mix (New England Biolabs) at 1× final concentration, 400 nM of each primer and 4 μl of the RT reaction, for a total volume of 10 μl. Real-time PCR was performed on a StepOnePlus instrument (Applied Biosystems), the amplification protocol consisting of a 10min step at 95°C for polymerase activation, followed by 40 cycles of 15s at 95°C and 60s at 60°C, and the melting curve step using instrument default settings. Results were analyzed by the ΔΔCT method (Pfaffl, 2001), using the rRNA subunit as normalizer. There were two hypothetical sequences on *P. brasiliensis* genome (ABKI00000000.2) (Desjardins et al., 2011), that could be responsible for DNase release: PADG_11161 (Gene ID: 22587058) and PADG_08285 (Gene ID: 22586608) (Access codes NW_011371358.1 and NW_011371372.1)33,38. The positive control was a fungal rRNA region (rRNA 5,8S). The primers were synthesized by Thermo Fisher Scientific – Brazil. The Real-Time RT-PCR from isolated neutrophil cultures was not performed as the presence of fungal primer sequences in the human genome was verified by GenBank, and no match was found. P. lutzii hypothetical regions for DNase expression PAAG_12429 and PAAG_07101 (Access code XM_015847918.1 and XM_002790819.1) were also tested in Pb18 and Pb265 samples, but no expression was detected (data not shown).

**Table 1:** Sequences of the primers used and their specificities.

Primer sequences		Product length	Target species
rRNA 5,8S		93 pb	Both
F:	TGAAGAACGCAGCGAAATGC		
R:	GGAATACCAGAGGGCGCAAT		
NW_011371358.1		146 pb	*P. brasiliensis*
F:	CCGGCAACAGCATTAGCATC		
R:	TGATCCGCTCTGATCTTCGC		
NW_011371372.1		127 pb	*P. brasiliensis*
F:	ACGACCGTCTCTTCCTCTCA		
R:	CGTTCAAAATCCTCGCTCGC		
XM_015847918.1		111 pb	*P. lutzii*
F:	TGCAGTTGAGATCCAATTACCCT		
R:	TGAACAAGGCCTCCCTTTGG		
XM_002790819.1		140 pb	*P. lutzii*
F:	TTCTTCAGGAGCTGCTACGC		
R:	TATCCGGCAGCAGTAAGACG		

### Statistics

All data were firstly tested by the Shapiro-Wilk normality test. Extracellular DNA quantification was analyzed by the Friedmann test, followed by the posttest of the multiple Dunn comparisons. Imaris analysis was tested by the Mann-Whitney test for fluorescence Intensity and paired T-test for Area. PCR analysis was performed by the Kruskal-Wallis test, followed by Dunn’s Test. Data were analyzed using GraphPad Prism 5.01 Software (GraphPad Software INC., CA, USA) with a significance value set at p<0,05%.

## Results

### Scanning Electron Microscopy of Neutrophil Cultures Challenged With Pb18 and Pb265 Isolates

**Figure 1:**
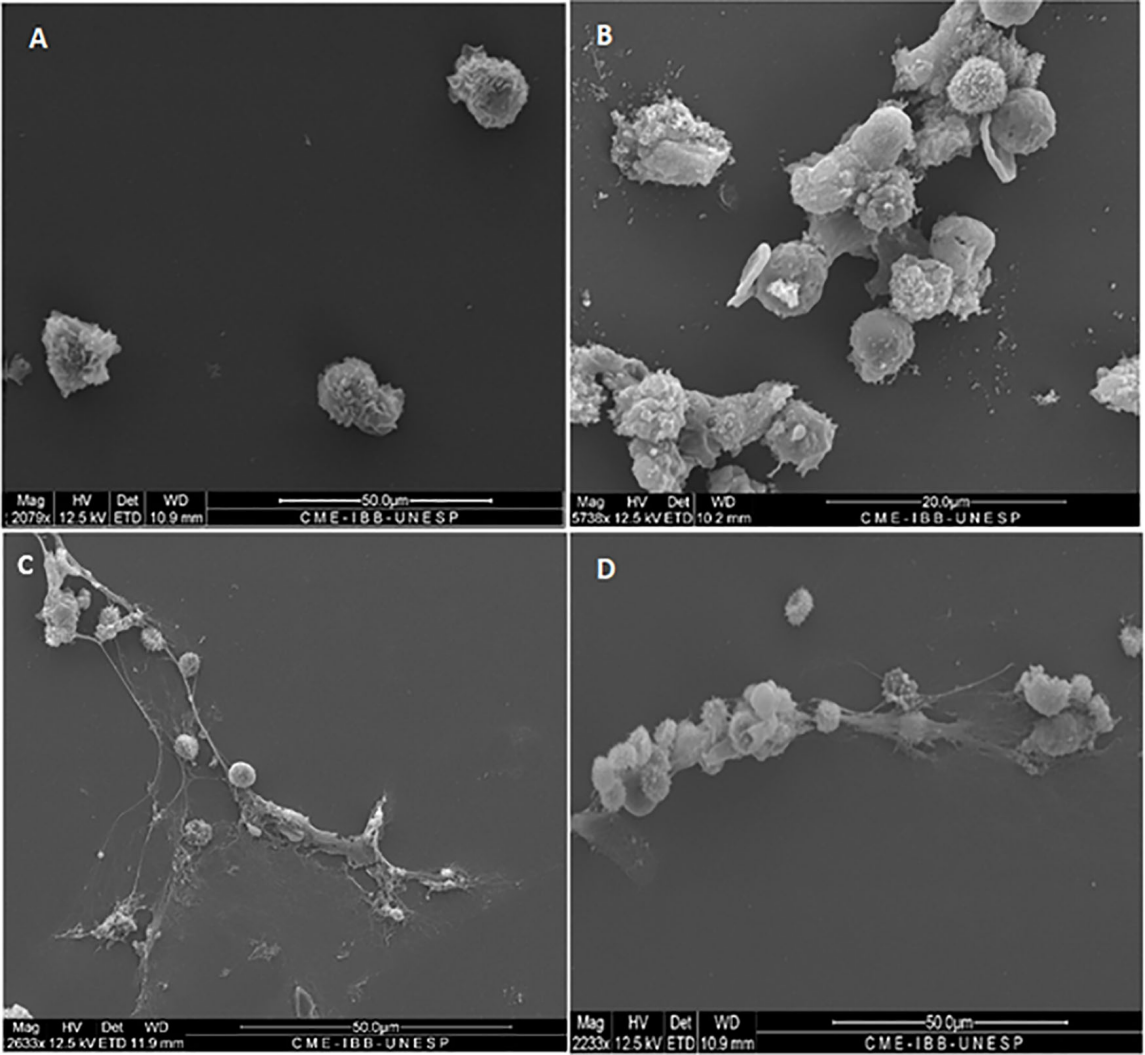
Scanning electron microscopy of neutrophils challenged or not with *P. brasiliensis* (50:1 ratio), showing different patterns of neutrophil extracellular traps (NETs) release **(C, D)**. All cultures were incubated during 2 h at 37°C at 5% CO2, with or not PMA+DNase (control cultures). **(A)** normal neutrophils; **(B)** neutrophils treated with PMA plus DNase, showing absence of NETs; **(C)** neutrophils challenged with Pb18 showing looser, dispersed and bigger aspect of NETs; **(D)** neutrophils challenged with Pb265 showing denser, more condensed and compacter aspect of NETs. The images are reprentative of 10 individuals tested.

PMN cultures were performed and challenged over 2 h, with two different isolates of *P. brasiliensis*, Pb18 and Pb265, and were analyzed by scanning electron microscopy. The images demonstrated that there were two different patterns of NETs for the two different isolates of the fungus, as showed in the previous study (Della Coletta et al., 2015). The neutrophil culture was incubated just with a culture medium for 2 h, a control culture (**Figure 1A**).

Neutrophil cocultures challenged with the Pb18 showed larger, looser, delicate, diffuse networks than NETs induced by Pb265, which covered most of the analysis area, consistent with a less effective structure in fungal entrapment (**Figure 1C**).

Unlike NETs released in response to the virulent isolate (Pb18), the NETs found in cultures challenged with the avirulent isolate (Pb265) were much more compact and denser, more localized, promoting higher yeast entrapment, as can be seen in the image (**Figure 1D**).

**Figure 1B** shows the absence of NETs, which were degraded by the action of DNase added in the cultures stimulated by PMA. It can also be observed some residues present in the slides, possibly from components present in the NETs, other than DNA, due to the degradation (**Figure 1B**).

### Confocal Microscopy of Neutrophil Cultures Challenged With Pb18 and Pb265 Isolates

**Figure 2:**
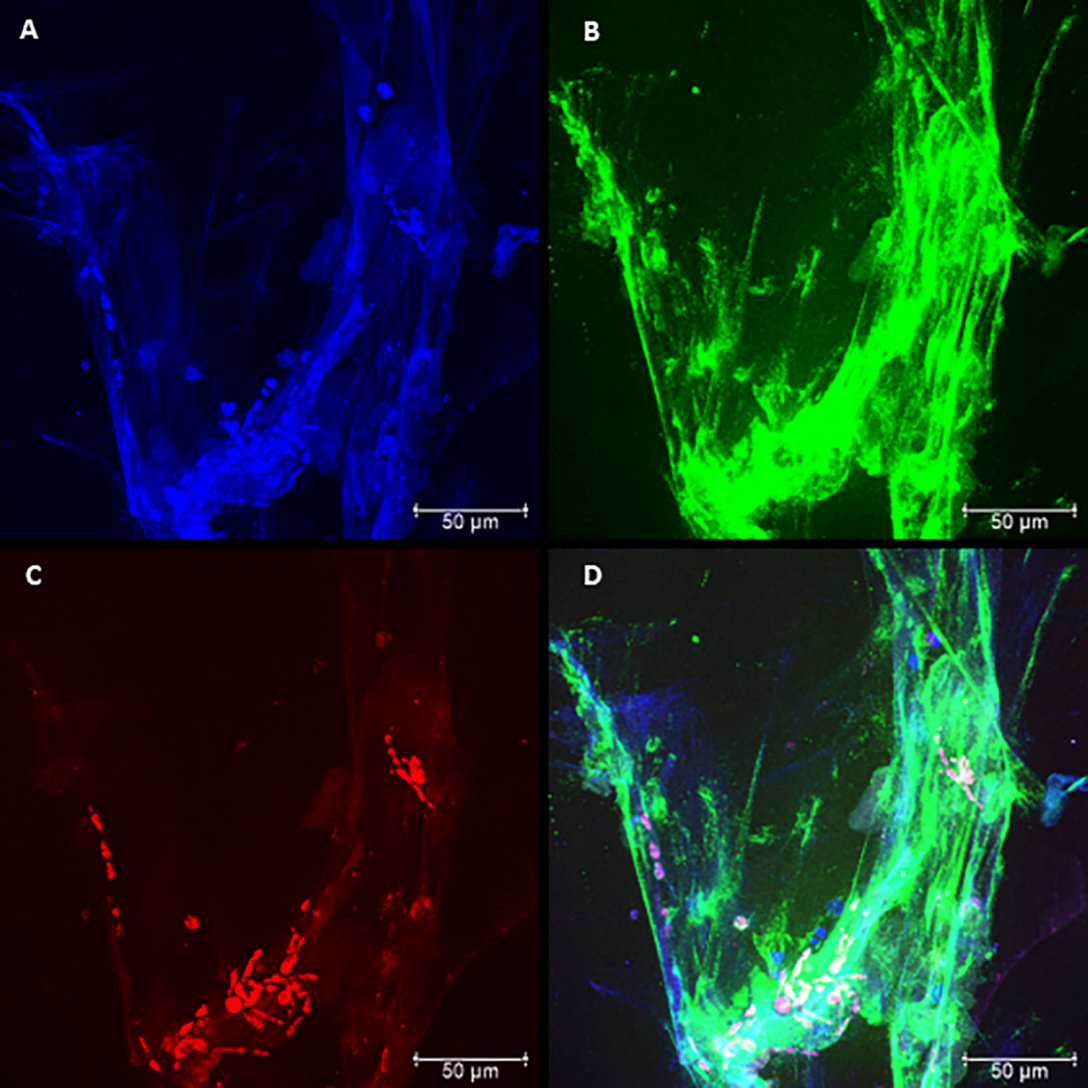
Confocal microscopy of neutrophils challenged with *P. brasiliensis* - Pb265 (50:1 ratio), showing the pattern of neutrophil extracellular traps (NETs) release. Cocultures were stained with DAPI **(A)**, labeled with anti-elastase antibody followed by FITC-conjugated secondary antibody **(B)**, and anti-histone H1 secondary antibody followed by Texas Red **(C)**. In the last frame, the overlapping images showing the three components of NETs **(D)**. (Bar size 50 μm). The images are reprentative of 10 individuals tested.

The presence of NETs and their components were evaluated by confocal microscopy, characterizing the presence of NETs in neutrophil cultures challenged with the avirulent fungal isolate (Pb265). Nuclear DNA and extracellular deconcentrated DNA were labeled with DAPI (**Figure 2A** and **Figure S1A**). Elastase and histones, other major components of NETs were also identified after immunostaining with specific primary and secondary antibodies (**Figures 2B, C** and **Figure S1B, C**) and, as expected, the overlap of the three stainings, showing the colocalization of three components (**Figure 2D** and **Figure S1D**). **Figure 2** and **Figure S1** show a dense, localized structures with very intense elastase immunostaining. It also caught our attention, the presence of fungi staining with anti-histone, allowing the identification of several fungi trapped in the formed NETs (**Figure 2C** and **Figure S1C**).

**Figure 3:**
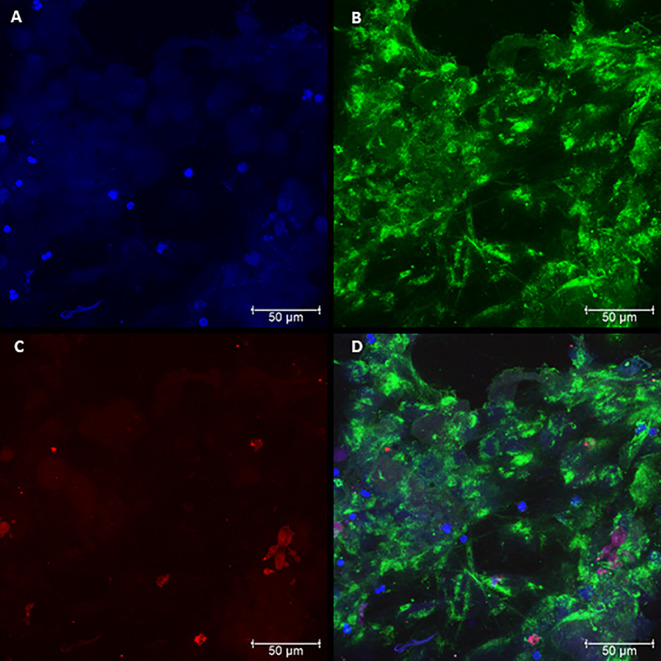
Confocal microscopy of neutrophils challenged with *P. brasiliensis* - Pb18 (50:1 ratio), showing the pattern of neutrophil extracellular traps (NETs) release. Cocultures were stained with DAPI **(A)**, labeled with anti-elastase antibody followed by FITC-conjugated secondary antibody **(B)**, and anti-histone H1 secondary antibody followed by Texas Red **(C)**. In the last frame, the overlapping images showing the three components of NETs **(D)**. (Bar size 50μm). The images are reprentative of 10 individuals tested.

By analyzing the slides of cultures challenged with Pb18, it was possible to see differences regarding the spatial arrangement and fluorescence intensity of these Pb18-induced NETs compared to Pb265-induced NETs. The virulent isolate Pb18 (**Figure 3** and **Figure S2**) induced the formation of a visually bigger, more dispersed, looking more discondensed and delicate structures with lower fluorescence intensity compared to the isolate Pb265 (**Figure 2** and **Figure S1**), whose induced denser, more compact, and with higher fluorescence intensity of NETs, as seen by electron scanning microscopy. Interestingly, it´s seems that histone staining (Texas RED) also marked the yeasts, although no specific fungal labeling was used (**Figure 3C** and **Figure S2C**), although the number of yeasts evidenced was lower. This fact makes apparent that in cultures challenged with Pb265 the number of yeasts trapped in NETs is much higher than in cultures challenged with Pb18.

### Neutrophil Extracellular Trap Quantification in Neutrophil Cultures Challenged With Pb18 and Pb265

**Figure 4:**
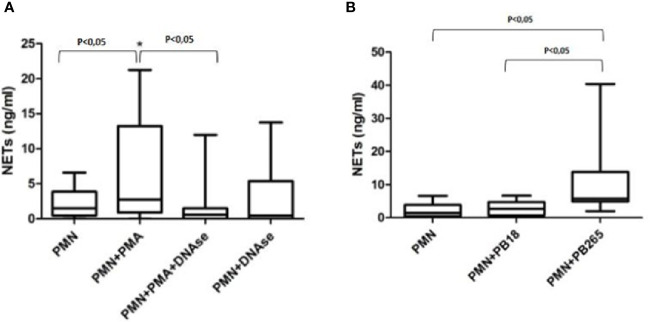
Quantification of neutrophil extracellular traps (NETs) in neutrophil culture supernatants treated or not with PMA (100 ng/ml) and/or DNase (100 U/ml) **(A)**; or in neutrophil culture supernatants challenged or not with Pb18 or Pb265 **(B)**. Representative box plots of 10 individuals tested.

The results shown that PMA-treated cultures showed a significant increase in the concentrations of NETs present in the cultures compared to normal neutrophils (**Figure 4A**). These values were significantly decreased when cultures were stimulated with PMA and treated with DNase, demonstrating that quantification was useful in identifying the increase in induction of NETs by PMA, and also decreased structures when stimulated cultures were treated with DNase, which promoted the degradation of the NETs.

The results regarding the quantifications of cultures challenged with Pb265 and Pb18 corroborate the data presented so far, demonstrating that the Pb265 induced a large amount of NETs compared to neutrophil-only or Pb18-challenged cultures (**Figure 4B**). The levels of NETs in cultures challenged with Pb18 demonstrate lower levels than Pb265-challenged cultures and could indicate a low capacity of this isolate to induce NETs or the action of a fungal DNase that would act on virulent isolate-induced NETs.

### Neutrophil Extracellular Trap Fluorescence Intensity and Area Quantification

**Figure 5:**
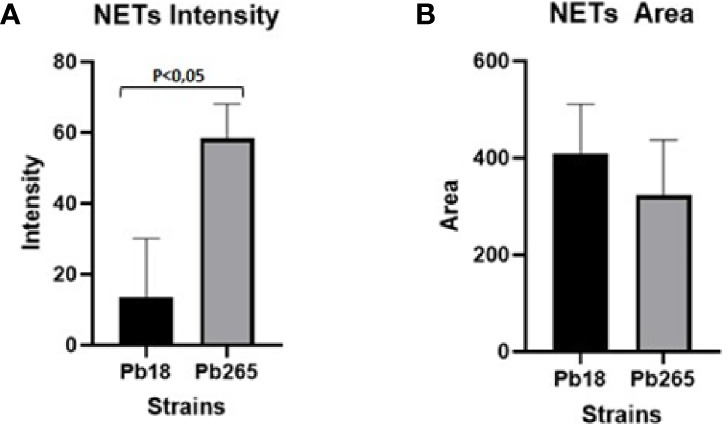
Analysis of neutrophil extracellular traps (NETs) fluorescence intensity **(A)** and area **(B)** by IMARIS software. The total area and intensity of the NETs were analyzed using the images acquired from cultures challenged with Pb18 and Pb265, using the elastase staining. The results are representative of aquired images of 10 individuals analysed.

The fluorescence intensity and area of NETs induced by the virulent and avirulent isolates of the fungus (Pb18 and Pb265, respectively), were evaluated using the confocal images acquired and the IMARIS^®^ software at Calvin, Phoebe, and Joan Snyder Institute for Chronic Diseases, University of Calgary/CA (**Figure 5**). Analysis showed that NETs released in response to the avirulent Pb265 were much more intense (fluorescence intensity) than those released in response to the virulent isolate (**Figure 5A**). However, the area of NETs was larger in virulent isolate images (**Figure 5B**), although the results did not show statistical differences between the two isolates.

### DNase Production by Fungi Evaluated by DNase Test Agar

**Figure 6:**
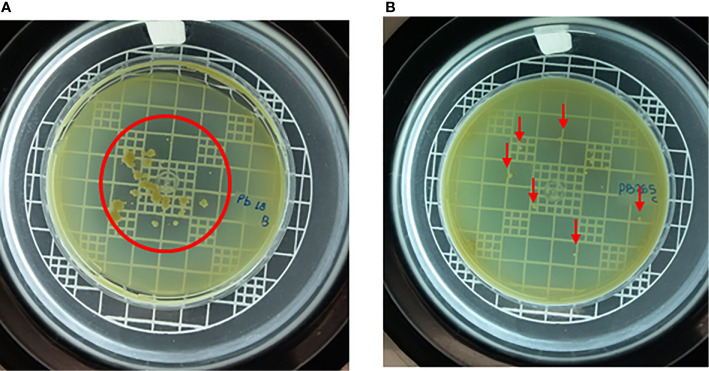
Virulent isolate (Pb18) **(A)** and avirulent isolate (Pb265) **(B)** grown on DNase test agar medium for 14 days, demonstrating degradation of DNA from medium by fungus. The red line marks the translucid halo boundaries in Pb18 plate, and the red arrow idicates the colonies of Pb265 without halo formation. Images representative of three experiments.

To assess the production and release of a possible DNase by the virulent and avirulent isolates of the fungus (Pb18 and Pb265, respectively), DNase Test Agar was used. The images made with the virulent isolate Pb18 demonstrated the degradation of the DNA present in the medium, leading to the formation of a halo around the fungal colonies. Because of the halos coalesced, we can spot a big translucent area (**Figure 6A**).

Cultivating the avirulent isolate in the DNase test agar medium, the images showed that the Pb265 isolate (**Figure 6B**) was not able to produce a halo as consistent and intense as the virulent isolate (Pb18). This fact corroborates our results regarding the previous analyzes. We conducted control experiments using a positive and negative control with *S. aureus* and *S. cerevisiae*, known as a DNase producer and non-DNase producer, respectively, to validate our analyses (**Figure S3**).

### DNase Expression Evaluation by RT-qPCR

To evaluate the gene expression over time for each isolate, we performed RT- qPCR. Pb18 and Pb265 were analyzed for both genes (PADG_11161 and PADG_08285) in isolated cultures and in PMN-fungus challenged cultures for 2 h.

**Figure 7:**
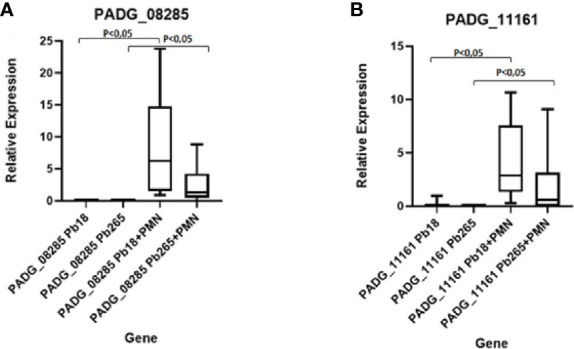
Relative Gene Expression of PADG_08285 and PADG_11161 Genes in neutrophil cultures challenged with Pb18 and Pb265. **(A)** Gene PADG_08285 and **(B)** Gene PADG_11161. Representative Box Plot from 10 individuals tested.

The Pb18 demonstrated a much higher relative expression of the PADG_08285 gene after 2 h of a challenge with neutrophil, when compared to the expression of the gene in the culture of isolated yeasts (**Figure 7A**). The Pb265 isolate also showed an increase in the relative expression of this gene after 2 h of challenge, higher than the isolated yeasts cultures. Therefore, the virulent isolate demonstrated a much higher expression of this gene than the avirulent isolate (**Figure 7A**).

The PADG_11161 gene also demonstrated higher relative expression in cultures of neutrophils challenged with both isolates than in isolated fungi cultures, whereas in cultures challenged with the virulent isolate, the expression was much higher than controls and cultures challenged with the avirulent isolate (**Figure 7B**).

## Discussion

The actions of NETs are studied in all sorts of inflammatory diseases - infection, sterile injury, cancer (Yipp and Kubes, 2013; Castanheira and Kubes, 2019; Liew and Kubes, 2019; Phillipson and Kubes, 2019; Snoderly et al., 2019; Mutua and Gershwin, 2020). Studies have shown that when the pathogen is too large to be phagocytized, neutrophils respond to releasing NETs by the process known as NETosis (Branzk et al., 2014). Other studies have already presented several microorganisms as NETs inducers, such as *S. aureus, Shigella flexneri, Streptococcus pneumoniae, Leishmania amazonensis, Aspergillus fumigatus, C. albicans, Aspergillus nidulans, and P. brasiliensis* itself, some of these studies even showing microbicidal activity (Brinkmann et al., 2004; Urban et al., 2006; Wartha et al., 2007; Bianchi et al., 2009; Bruns et al., 2010; Brinkmann and Zychlinsky, 2012; Mejía et al., 2015; Della Coletta et al., 2015; Bachiega et al., 2016; Thanabalasuriar et al., 2019; Castanheira and Kubes, 2019; Liew and Kubes, 2019).

The interaction between *P. brasiliensis* and NETs is still being investigated, but a study has already shown the production of NETs in contact with *P. brasiliensis* yeast and how these structures are involved in extracellular fungal death, also showing that the dectin-1 receptor mainly mediates the NETs released in response to the *P. brasiliensis* isolates (Bachiega et al., 2016). The NETs release against *P. brasiliensis* have also been demonstrated in cutaneous lesions in patients with paracoccidioidomycosis (Della Coletta et al., 2015), corroborating with the previous studies. The same study that raised the hypothesis that distinct NETs patterns induced by different isolates of the fungus (Pb18 and Pb265), could be related to a fungal DNase-like activity, once the enzyme (DNase) is an endonuclease that catalyzes the hydrolysis of extracellular DNA (Lazarus and Wagener, 2019).

In this study, the Scanning Electron Microscopy assays demonstrated similar structures to those shown in the previous studies (Della Coletta et al., 2015; Bachiega et al., 2016). In the current results, the release of NETs was evidenced and confirmed by confocal microscopy, where it was observed the presence and colocalization of extracellular DNA, histones, and elastase. However, the patterns of the NETs differed from each other according to the fungal isolate tested. When the most virulent isolate (Pb18) was evaluated, the NETs aspect was looser, dispersed, covering most area of the coverslips. When the less virulent isolate (Pb265) was tested, the NETs were denser, more condensed and compacter, covering smaller areas of the coverslips, closer to the fungus. These results directly resemble previous studies (Della Coletta et al., 2015). The difference between NETs was also demonstrated by the analysis of intensity using IMARIS software and by quantification method. The results showed that the fluorescence intensity of NETs released in response to Pb265 was greater than that released in response to Pb18. These results suggest a possible degradation of the DNA present in the NETs by the virulent isolate, corroborating the hypothesis mentioned by Della Coletta et al. (2015), that Pb18 isolate could be producing some protein with DNase-like activity and that this protein could be degrading the NETs, acting as an escape mechanism (Della Coletta et al., 2015), or even a lower induction capacity of NETs by this isolate. Some studies have already shown the release of endonucleases by different microorganisms as an escape mechanism from NETs, as already indicated (Buchanan et al., 2006; Berends et al., 2010; Sánchez and Colom, 2010; Thammavongsa et al., 2013; Seper et al., 2013; de Buhr et al., 2014; Morita et al., 2014; Bentubo and Gompertz, 2014; Riceto et al., 2015; Zhang et al., 2016; Gondaira et al., 2017; Liu et al., 2017; Teymournejad et al., 2017; Zhang et al., 2017; Hoppenbrouwers et al., 2018; Wilton et al., 2018; Mitiku et al., 2018; Sharma et al., 2019; Freitas-Mesquita et al., 2019), and such as some pathogenic fungi such as *C. albicans* (Riceto et al., 2015; Zhang et al., 2017)*, Cryptococcus spp* (Sánchez and Colom, 2010) and*, Trichosporon spp* (Bentubo and Gompertz, 2014). These microorgansms use these nucleases as a way of cleaving NETs, enhancing their survival within their hosts.

Interestingly, although no specific labeling was used for the fungus yeast, we noted that the histone labeling was able to label *P. brasiliensis* yeasts, enabling the identification of yeasts trapped in the formed NETs. This fact, although not expected, allowed very interesting observations, demonstrating that in the images related to cultures challenged with Pb265, there were a much larger number of yeasts that were trapped in the formed NETs, a fact that was not observed in cultures challenged with isolate Pb18, that is, the presence of trapped fungus was much smaller when compared to cultures challenged with avirulent isolate, a phenomenon that could be explained by our results. And another observation is that if the yeasts were labeled with anti-histone, allowing the visualization of the fungus in the images, probably was due to an effect of yeast coverage by histones present on the NETs, that could be a factor involved in fungicidal activity of NETs against fungi, once it was demonstrated previously by our research group, that NETs can act against *P. brasiliensis*, causing yeast morphological changes and fungus killing (Bachiega et al., 2016). Besides histones activities on binding and regulating DNA expression, they also exert defensive functions and promote the inflammatory response, being the major components of NETs, ​​contributing both to the killing of pathogens and to tissue injury (Hoeksema et al., 2016). Ballard et al. (2020) (Ballard et al., 2020) demonstrated that lysozyme and histones inhibited hyphal metabolic activity in *A. fumigatus* isolates in a dose-dependent manner, and imaging flow cytometry revealed that histones inhibited the germination of *A. fumigatus* conidia. Recently, it was demonstrated that histone H2A enters *Escherichia coli and S. aureus* through membrane pores formed by the AMPs LL-37 and magainin-2, depolarizing the bacterial membrane potential, and impairing membrane recovery (Doolin et al., 2020). Once inside cell, H2A reorganizes bacterial chromosomal DNA, inhibiting global transcription, acting directly on killing of bacteria (Doolin et al., 2020).

The presence of NETs was also evaluated and quantified by an immunoassay using anti-elastase and Quant-IT Picogreen Kit, and this methodology has already been used in other studies (Czaikoski et al., 2016; Colón et al., 2019). The extracellular DNA quantification was used to assess the NETs release in response to *Mycobacterium tuberculosis*, *L. amazonensis* and other microorganisms as well (Ramos-Kichik et al., 2009; Guimarães-Costa et al., 2009; Della Coletta et al., 2015; Bachiega et al., 2016). However, the use of the Picogreen Kit alone, with only extracellular DNA labeling, has been widely criticized, since extracellular DNA could come from both the NETosis process and other cellular phenomena. Thus, we chose to quantify the elastase-extracellular DNA complex, giving us the quantification of NETs. Our results showed that the culture challenged with the virulent isolate presented a smaller amount of NETs when compared to the avirulent isolate, corroborating the confocal and scanning microscopy results, and the above results.

As said before, some organisms are capable of releasing DNase to escape from the immune system (Buchanan et al., 2006; Berends et al., 2010; Sánchez and Colom, 2010; Thammavongsa et al., 2013; Seper et al., 2013; de Buhr et al., 2014; Morita et al., 2014; Bentubo and Gompertz, 2014; Riceto et al., 2015; Zhang et al., 2016; Gondaira et al., 2017; Liu et al., 2017; Teymournejad et al., 2017; Zhang et al., 2017; Hoppenbrouwers et al., 2018; Wilton et al., 2018; Mitiku et al., 2018; Sharma et al., 2019; Freitas-Mesquita et al., 2019) and the DNase Test Agar is commonly used in tests to identify this production, mainly by *S. aureus*, since this microorganism is a known producer of DNase (Kateete et al., 2010). In the present study, using this methodology, it was possible to identify the production of a DNase-like protein by the virulent isolate of *P. brasiliensis* (Pb18), demonstrating DNA degradation in the cultures plaques, while the avirulent isolate did not demonstrate any significant production nor DNase activity. This result prove the hypothesis raised in our previous studies, which showed that the NETs released in response to the virulent isolate were looser and had a more degraded appearance when compared to the NETs released in response to the avirulent isolate (Della Coletta et al., 2015). This leads us to believe that this pattern of NETs occurs due to a release of a protein with DNase-like activity by the virulent isolate, as shown by our results, that Pb18 could produce a DNase-like protein, suggesting an action on NETs degradation, which would explain the observed results.

With the identification of a DNase-like protein action by Pb18 isolate using DNase Test Agar, it was tested different genes (PADG_08528 and PADG_11161) that was found in the fungus genome as hypothetical proteins (Desjardins et al., 2011), and it was demonstrated that the PADG_08528 gene showed much higher expression than the PADG_11161 gene when yeast cells were challenged with human neutrophils. Interestingly, both genes showed higher expression in Pb18 yeast challenged cultures. This demonstrates that both genes may be associated with the production and release of a DNase-like, however when these yeasts were placed in contact with human neutrophils, the PADG_08528 gene had a much larger expression, thus, probably, indicating that this gene could be involved in the release of the DNase-like protein and consequent degradation of NETs. These results also confirm the raised hypothesis, that the virulent isolate (Pb18) would be releasing a DNase-like protein as a way to degrade NETs and survive the neutrophils attack (Della Coletta et al., 2015).

Thus, given the observed results we can conclude that the NETs pattern induced by the virulent isolate (Pb18) with looser, dispersed and bigger aspect is due the release of a DNase-like protein by Pb18. This factor could be an important escape mechanism of the fungus to circumvent the action of NETs, thus subverting this important neutrophil effector mechanism. Our lab is conducting other studies to better identify and characterize this protein by these isolates and other species of *Paracoccidioides.*

## Data Availability Statement

The raw data supporting the conclusions of this article will be made available by the authors, without undue reservation.

## Ethics Statement

The studies involving human participants were reviewed and approved by the Research Ethics Committee of Medical School of Botucatu, UNESP – São Paulo State University (CAAE: 85654018.9.0000.5411; protocol: 2.577.243/2018). The patients/participants provided their written informed consent to participate in this study.

## Author Contributions

LADM conceptualized the study. YRZ, ALOD, AMDC, LC, KSTY, LAS, ICD, RMK, MEW, and VFX developed the methodology. YRZ, ALOD, AMDC, LC, KSTY, LAS, ICD, and RMK validated the study. YRZ, ALOD, AMDC, LC, KSTY, LAS, ICD, and RMK conducted the investigation. LADM, YRZ, LAS, ICD, and RMK performed the formal analysis. LADM provided the resources. LADM and YRZ wrote the original draft. LADM, RMK, LZ, and PK wrote, reviewed, and edited the manuscript. LM supervised the study. LM conducted the project administration. All authors contributed to the article and approved the submitted version.

## Funding

This study was supported by São Paulo Research Foundation (FAPESP 2018/09706-7) and FAPESP MS fellowship (YRZ—FAPESP 2017/26230-3). This study was financed in part by the Coordenação de Aperfeiçoamento de Pessoal de Nível Superior—Brasil (CAPES)—Finance Code 001, master student in the Post-Graduate Program in Pathology, Botucatu Medical School, UNESP—São Paulo State University, Botucatu, São Paulo, Brazil. It was also supported by CAPES PrInt: Program for Institutional Internationalization granted to LAS, and by Programa de Apoio para a realização de Estágio no Exterior (PAREex)—PROPG UNESP—Edital 03/2018 PROPG, granted to LADM, head of the Department of Pathology, Botucatu Medical School, UNESP—São Paulo State University, Botucatu, São Paulo, Brazil. The funders had no role in study design, data collection, and analysis, decision to publish, or preparation of the manuscript.

## Conflict of Interest

The authors declare that the research was conducted in the absence of any commercial or financial relationships that could be construed as a potential conflict of interest.
