# Supplementary material for: Paracoccidioides brasiliensis Releases a DNase-Like Protein That Degrades NETs and Allows for Fungal Escape
Source: Front Cell Infect Microbiol. 2021 Feb 10;10:592022. doi: 10.3389/fcimb.2020.592022 (PMC7902888; doi:10.3389/fcimb.2020.592022)
Supplement: Supplementary file 1 [file DataSheet_1.pdf]

Supplemental material

S1

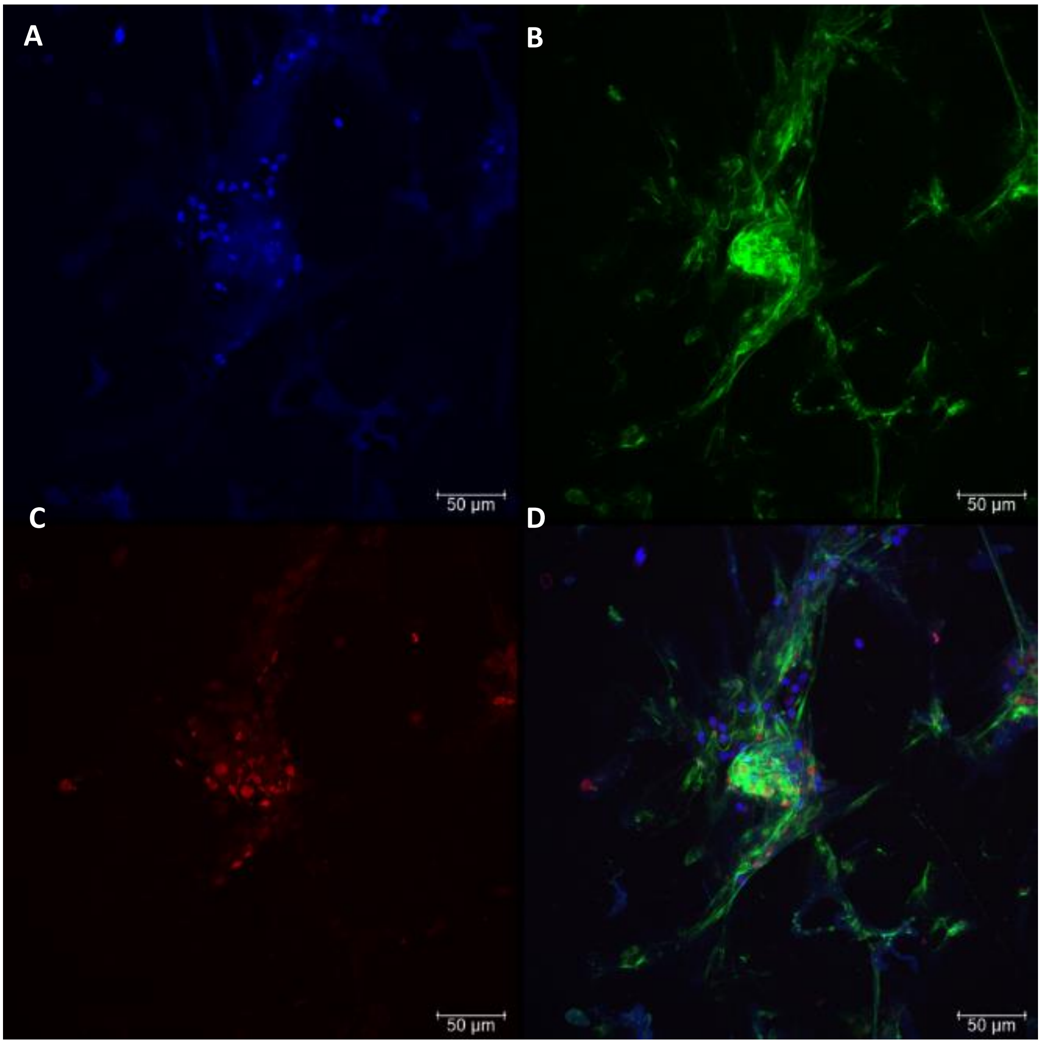

S1. Confocal microscopy of neutrophils challenged with *P. brasiliensis* Pb265 (50:1 ratio), showing the pattern of NETs release. Cocultures were stained with DAPI (A), labeled with anti-elastase antibody followed by FITC-conjugated secondary antibody (B), and anti-histone H1 secondary antibody followed by Texas Red (C). In the last frame, the overlapping images showing the three components of NETs (D). (Bar size 50µm).

S2

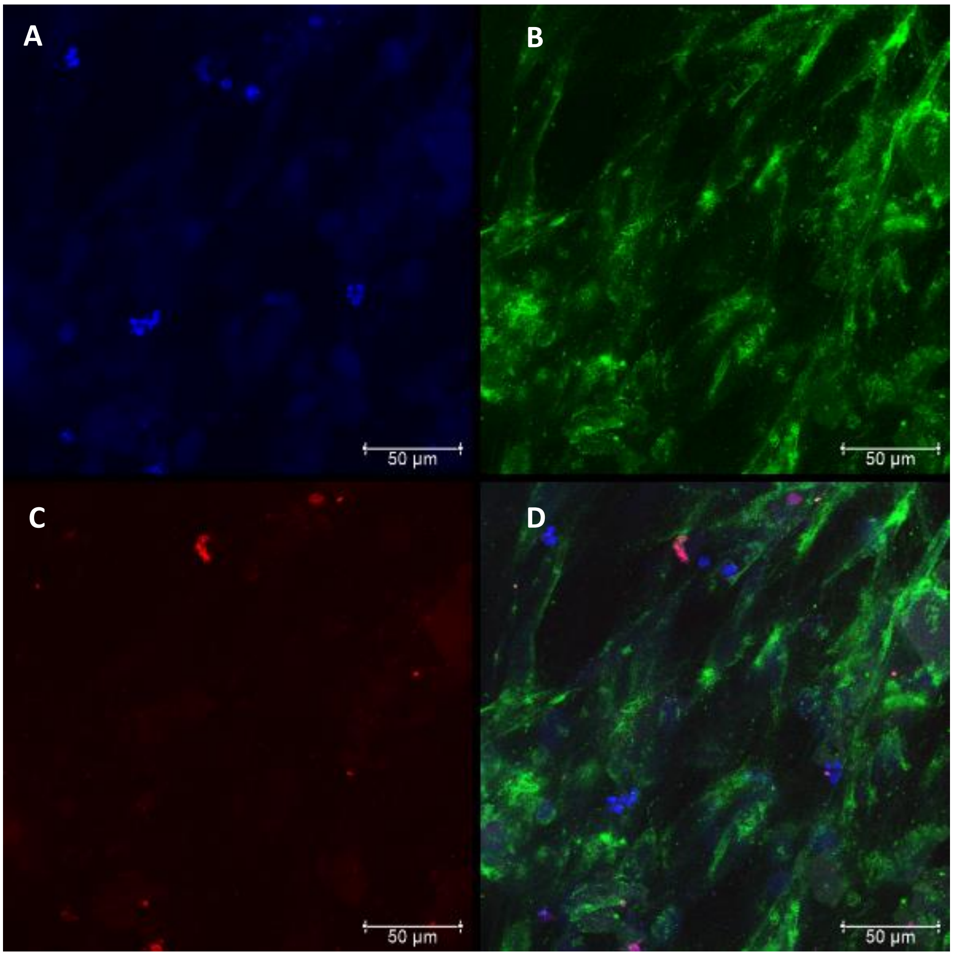

S2. Confocal microscopy of neutrophils challenged with *P. brasiliensis* Pb18 (50:1 ratio), showing the pattern of NETs release. Cocultures were stained with DAPI (A), labeled with anti-elastase antibody followed by FITC-conjugated secondary antibody (B), and anti-histone H1 secondary antibody followed by Texas Red (C). In the last frame, the overlapping images showing the three components of NETs (D). (Bar size 50µm).

S3

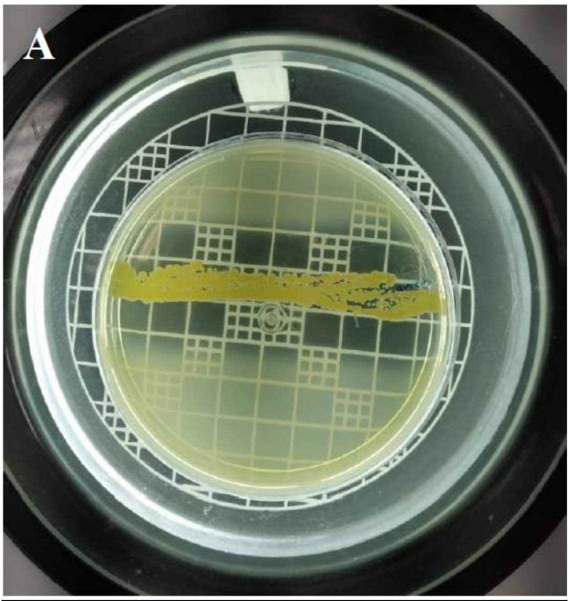

S3. *S. aureus* grown on DNAse test agar medium for 1 day, demonstrating degradation of DNA from medium by bacterial DNAse, showing the translucid halo formed. Images representative of three experiments.
